# Pericarotid fat density: a novel predictor of cognitive and functional outcomes in cerebral small vessel disease

**DOI:** 10.3389/fnagi.2026.1832953

**Published:** 2026-08-03

**Authors:** Shuo Zhao, Jing Zhang, Xunyao Hou, Xinyao Wang, Hui Gu, Chuanchen Zhang, Yunliang Guo, Ximing Wang

**Affiliations:** 1 Department of Radiology, Shandong Provincial Hospital Affiliated to Shandong First Medical University, Jinan, China; 2 Department of Radiology, Liaocheng People’s Hospital, Shandong First Medical University & Shandong Academy of Medical Sciences, Liaocheng, China; 3 Department of Radiology, Shandong Provincial Hospital, Cheeloo College of Medicine, Shandong University, Jinan, China; 4 Shandong Key Laboratory of Multimodal Imaging Artificial Intelligence and Genetics, Jinan, China; 5 Department of Geriatric Neurology, Shandong Provincial Hospital Affiliated to Shandong First Medical University, Jinan, China; 6 Department of Geriatrics, Shandong Provincial Hospital Affiliated to Shandong First Medical University, Jinan, China

**Keywords:** cerebral small vessel disease, cognitive impairment, computed tomography angiography, event-related potential, pericarotid fat density

## Abstract

**Background:**

Pericarotid fat density (PFD) obtained by computed tomography angiography (CTA) could serve as a surrogate biomarker reflecting local inflammation. The study aimed to quantitatively estimate the impact of PFD on cognitive and functional outcomes in patients with cerebral small vessel disease (CSVD).

**Methods:**

A total of 71 symptomatic and 55 asymptomatic CSVD patients were prospectively recruited. Imaging characteristics comprising CSVD markers and carotid artery markers (PFD values, calcification, stenosis degree, ulceration, and maximum wall thickness) were analyzed. Cognitive assessments were performed 90 days after acute infarcts, including diverse neuropsychological scales (the Montreal Cognitive Assessment, the Shape Trail Test, the Stroop test, and the Rey–Osterrieth Complex Figure Test) and sensitive event-related potential (ERP) detection. Worse functional outcomes were defined as a Modified Rankin Scale change score (ΔmRS) ≥ 0 (ΔmRS = mRS_90-day_−mRS_baseline_) in the symptomatic CSVD cohort.

**Results:**

Symptomatic CSVD showed significantly higher PFD values than asymptomatic CSVD. As indicated by multiple scales and P3a/P3b amplitude, cognitive function in symptomatic CSVD was poorer than that in asymptomatic CSVD. Symptomatic CSVD showed numerically stronger correlations between PFD values and cognitive impairment, with more parameters involved and the strongest correlations existing among ERP data. Maximum PFD was identified as an independent predictor of adverse functional outcomes (*p* < 0.001), and a predictive model incorporating maximum PFD showed better performance (AUC = 0.939).

**Conclusion:**

The increased PFD values were closely associated with cognitive impairment and adverse functional outcomes in CSVD patients, especially among those with symptoms. Our findings could assist in the risk stratification and tailored treatment of CSVD patients to facilitate prognosis.

## Highlights

- An increased pericarotid fat density (PFD) was significantly correlated with cognitive impairment and unfavorable functional outcomes at 90 days in cerebral small vessel disease (CSVD), especially among those with symptoms.
- The incorporation of PFD improved the predictive performance of the constructed model for poor functional outcomes in CSVD patients.
- PFD obtained by computed tomography angiography (CTA) provides additional predictive value for CSVD in risk stratification and early targeted therapeutics.

## 1 Introduction

Cerebral small vessel disease (CSVD) denotes a clinical and imaging syndrome involving small vessels in the brain due to the presence of various risk factors (Charidimou et al., 2016). CSVD contributes to approximately 20–30% of ischemic strokes, as well as up to 45% of dementia cases. Radiologically, the main manifestations of CSVD comprise recent small subcortical infarcts (RSSIs), lacunes, white matter hyperintensities (WMHs), cerebral microbleeds (CMBs), enlarged perivascular spaces (EPVS), and brain atrophy (Wardlaw et al., 2013). Some CSVD patients show overt clinical symptoms, which are primarily characterized by lacunar syndromes and RSSIs (lacunar infarcts) on magnetic resonance imaging (MRI), while others have no symptoms or sometimes mild symptoms, including dizziness and an unbalanced gait (Cannistraro et al., 2019).

Symptomatic CSVD is characterized by heterogeneous clinical manifestations and various prognostic outcomes (Wardlaw et al., 2019; Tao et al., 2022); hence, early identification of predictors associated with unfavorable outcomes is essential for risk stratification and clinical management. Characterized by an insidious onset, cognitive impairment is a long-lasting and severe complication of CSVD (Pinter et al., 2015; Elahi et al., 2023). In addition to traditional neuropsychological scales, e.g., the Montreal Cognitive Assessment (MoCA) and the Brief Memory and Executive Test (Peng, 2019; Nannoni et al., 2022), event-related potential (ERP) is an emerging and highly sensitive tool used in neurological disorders to detect mild cognitive impairment (Helfrich and Knight, 2019). P3 is the most extensively studied ERP component and is known to reflect attentional allocation and decision-making processes during tasks (Fjell et al., 2007). To date, no study has integrated an ERP task with multiple neuropsychological assessments to comprehensively evaluate cognitive function in both symptomatic and asymptomatic CSVD patients.

Inflammation is closely associated with the development and progression of CSVD lesions (Evans et al., 2021; Zhang D. D. et al., 2022; Zhang S. et al., 2022). Compared with conventional laboratory examinations (e.g., serum inflammatory markers), precise identification of local perivascular inflammation may be more efficient in guiding individualized prevention and therapy for devastating cerebrovascular events (Besler et al., 2024; Cau et al., 2024; Xu et al., 2025). The perivascular adipose tissue (PVAT) adjacent to the carotid artery contains inflammatory mediators, and inflammatory changes occurring in PVAT are considered to play critical roles in the pathogenesis of cerebrovascular diseases (Qi et al., 2018). Convergent evidence has suggested that increased pericarotid fat density (PFD) obtained by routine CTA could serve as a surrogate biomarker to reflect PVAT inflammation, and correlations between PFD and vulnerable carotid plaques were reported (Zhang S. et al., 2021; Yu et al., 2022), which may lead to the occurrence of cerebrovascular symptoms or infarcts (Baradaran et al., 2018; Zhang S. et al., 2022). In previous retrospective studies, PFD values were strongly associated with neuroimaging markers, disease progression, and functional outcomes in CSVD (Zhang D. H. et al., 2021; Fu et al., 2025; Zhang et al., 2025). Nevertheless, to the best of our knowledge, the performance of PFD in predicting cognitive outcomes in CSVD remained unknown.

The present study aimed to quantitatively estimate pericarotid fat tissue inflammation evaluated by PFD on CTA and determine its impact on cognitive impairment in CSVD patients. In addition, we aimed to identify the correlation between PFD and functional outcomes in the subset of symptomatic patients.

## 2 Materials and methods

This prospective study was approved by the Institutional Review Board of Shandong Provincial Hospital Affiliated to Shandong First Medical University (SWYX: No. 2024-072) and conformed to the ethical criteria of the Declaration of Helsinki. Written informed consent was obtained from all participants prior to the study.

### 2.1 Participants and criteria

**Figure 1:**
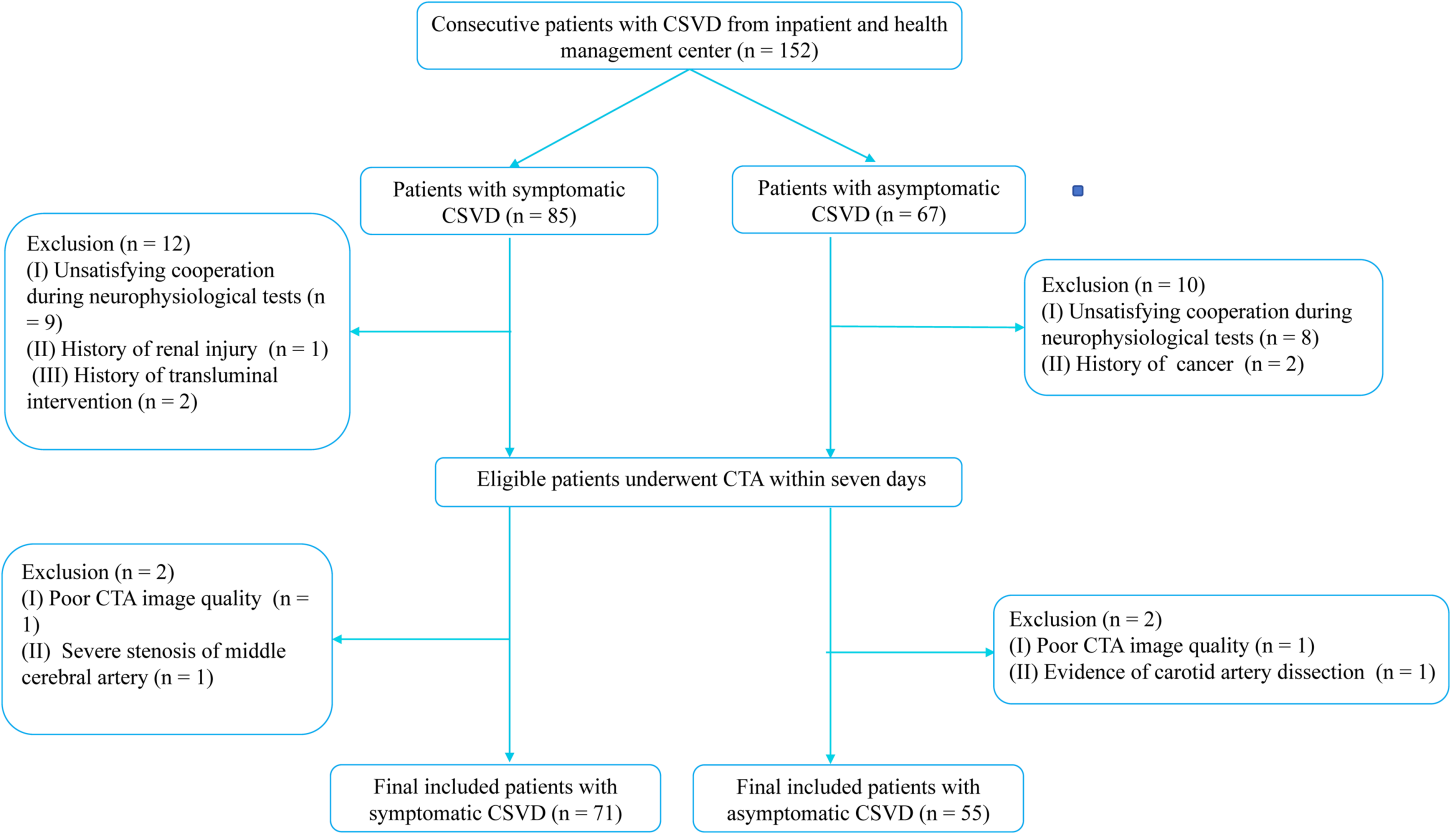
The flowchart of inclusion and exclusion for symptomatic and asymptomatic CSVD patients. CSVD, cerebral small vessel disease.

From January 2024 to April 2025, we consecutively and prospectively recruited patients diagnosed with CSVD from the inpatient department and health management center. All patients underwent carotid CTA and brain MRI scans. As shown in Figure 1, patients were included if they met the following criteria: (1) symptomatic CSVD with clinical lacunar syndromes and anatomically corresponding RSSIs confirmed on MRI (Nannoni et al., 2022); (2) asymptomatic CSVD without overt symptoms and with typical radiological features, including lacunes, WMHs, CMBs, EPVS, or brain atrophy (Wardlaw et al., 2013); (3) age range of 35 to 75 years; and (4) adequate cooperation (ERP task accuracy ≥90%) and stable condition during examinations. Patients were excluded if they had the following criteria: (1) allergy to iodine contrast media or a history of renal dysfunction (glomerular filtration rate <60 mL/min/1.73 m^2^); (2) extracranial or intracranial macrovascular stenosis >50%; (3) intracranial hemorrhage; (4) cardiogenic infarct; (5) non-CSVD-related WMHs, such as multiple sclerosis; (6) were comorbid with other neurological disorders, such as Parkinson’s disease; (7) were comorbid with systemic diseases, such as tumors; (8) moderate to severe anxiety, depression, or somatic symptoms; (9) medication usage that might influence cognition; (10) visual/hearing impairment or illiterate; (11) history of transluminal intervention; and (12) non-atherosclerotic cerebrovascular diseases, such as arterial dissection.

The clinical, imaging, and cognitive data were obtained. The National Institutes of Health Stroke Scale (NIHSS), Modified Rankin Scale (mRS), and activities of daily living (ADL) assessment (Barthel Index) were performed to evaluate neurological impairment, global function, and daily living status, respectively.

### 2.2 Image acquisition and analysis

CTA and MRI images were independently evaluated by specialized radiologists who were experienced in vascular imaging. They were blinded to the patients’ identities, and any discrepancies were settled by consensus.

#### 2.2.1 Brain MRI

Brain MRI was performed using a 3.0T MRI scanner (Magnetom Prisma, Siemens Healthineers, Germany) with a standard 32-channel head coil. The examination parameters were as follows: time to repetition (TR)/time to echo (TE) = 2000 ms/7.4 ms and slice thickness (ST) = 5.0 mm for T1-weighted imaging (T1WI); TR/TE = 4,300 ms/109 ms and ST = 5.0 mm for T2-weighted imaging (T2WI); TR/TE = 8,000 ms/81 ms and ST = 5.0 mm for fluid-attenuated inversion recovery (FLAIR); TR/TE = 2,680 ms/57 ms and ST = 1.5 mm for diffusion-weighted imaging (DWI); and TR/TE = 27 ms/20 ms and ST = 1.5 mm for susceptibility-weighted imaging (SWI).

Neuroimaging characteristics of CSVD were assessed in both hemispheres according to the STandards for ReportIng Vascular changes on nEeuroimaging (STRIVE) consensus criteria (Wardlaw et al., 2013). RSSIs were defined as lesions with a maximum diameter of <20 mm (axial position) with high signal intensity on DWI. Lacunes appeared as signals similar to cerebrospinal fluid on T1WI and T2WI, with hyperintensity surrounding the rims on FLAIR (diameters of 3–15 mm). WMHs were evaluated using the Fazekas visual rating scale, including paraventricular grades (0, absence; 1, cap; 2, halo; 3, extending into deep white matter) and deep grades (0, absence; 1, punctate; 2, beginning confluence; 3, extensive confluence). CMBs showed small hypointense signals with diameters of 2–10 mm on SWI. EPVS were defined as round or linear fluid-filled cavities with a diameter of <3 mm and were located in the basal ganglia (0 points, absence; 1 point, <10; 2 points, 11–20; 3 points, 21–40; 4 points, >40).

The CSVD total score combines four neuroimaging parameters, with an ordinal scale ranging from 0 to 4. One point was allocated for the presence of the following manifestations: (1) ≥1 lacunes; (2) periventricular WMH Fazekas grade 3 or deep WMH Fazekas grades ≥2; (3) ≥1 CMBs; and (4) moderate to severe EPVS (≥2 points) in the basal ganglia.

#### 2.2.2 Carotid CTA

Carotid CTA was performed using a third-generation dual-source CT scanner (SOMATOM Force, Siemens Healthineers, Germany) with the following parameters: a tube voltage of 80 kV or 100 kV based on the body mass index (≤25 kg/m^2^, 80 kV; >25 kg/m^2^, 100 kV), a pitch of 1.0, a rotation time of 0.35 s, a reconstructed slice thickness of 1.5 mm, and a slice interval of 1.0 mm. Iodinated contrast material (60–80 mL; Omnipaque 350, GE Healthcare) was injected at a flow rate of 4 mL/s, followed by 40 mL of normal saline at the same flow rate. Bolus tracking was used to trigger image acquisition 5 s after the aortic arch reached an attenuation threshold value of 100 HU.

Imaging characteristics of the carotid artery were evaluated on a dedicated postprocessing workstation (Syngo.Via, Siemens Healthineers, Germany), including calcification, stenosis degree, ulceration, and maximum wall thickness. The extracranial carotid artery stenosis degree, incorporating the internal carotid artery (ICA), external carotid artery, and common carotid artery, was analyzed according to the North American Symptomatic Carotid Endarterectomy Trial (NASCET) criteria (Barnett et al., 1991). Patients were divided into three grades based on the inclusion criteria: non-stenosis, 0–29% stenosis, and 30–49% stenosis. Maximum wall thickness was measured on the maximal cross-section of the plaque that was perpendicular to the long axis of the vessel. Plaque ulceration was defined as contrast media extending at least 2 mm into the plaque (Jean Marie et al., 2010).

#### 2.2.3 Quantification of PFD

**Figure 2:**
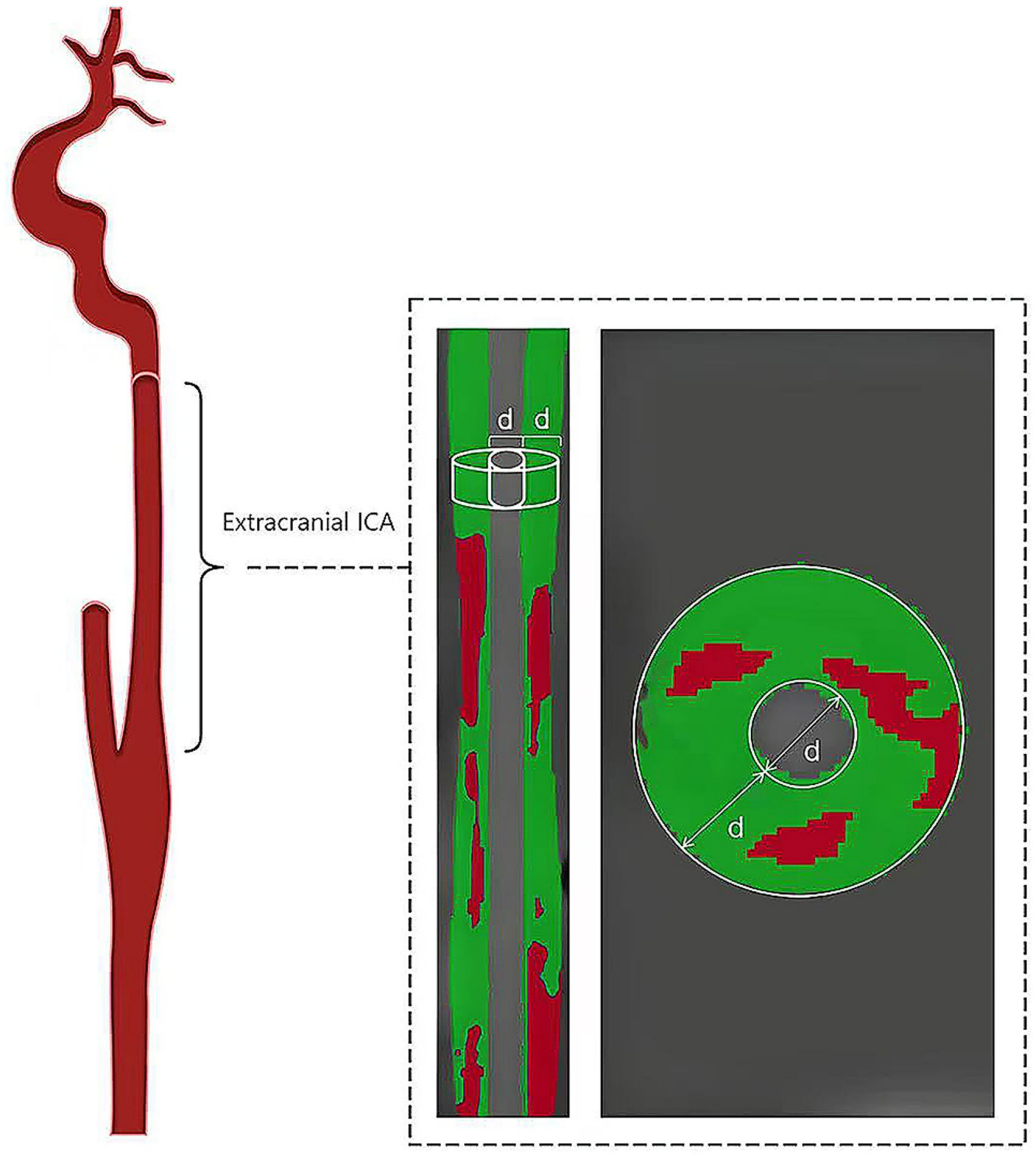
The analytical details of pericarotid fat density. Perivascular fat density was analyzed by measuring adipose tissue surrounding the extracranial internal carotid artery (ICA), within a radial distance equivalent to the diameter (*d*) of the vessel.

PVAT attenuation surrounding the extracranial ICA was quantified using dedicated software (Perivascular Fat Analysis Tool, Shunkun Technology, Beijing, China). An established measurement previously described in pericoronary fat was used (Oikonomou et al., 2018). PFD surrounding the total extracranial ICA was semi-automatically evaluated in the form of voxels with a density range between −190 HU and −30 HU, and the radial distance from the outer vessel wall was equal to the average diameter of the target vessel. Accordingly, the mean and maximum PFD of the bilateral extracranial ICA were analyzed. The analytical details of PFD are provided in Figure 2.

### 2.3 Cognitive and emotional assessments

Cognitive evaluations were performed 90 days after onset in symptomatic patients to minimize the influence of acute-phase infarcts.

#### 2.3.1 Neuropsychological tests

Cognitive function was assessed using the following instruments: MoCA for general cognitive screening (Tian et al., 2020); the Stroop test for measuring executive function, selective attention, and conflict monitoring (Scarpina and Tagini, 2017); the Shape Trail Test (STT) for evaluating attention, cognitive flexibility, and hand-eye coordination (Wang et al., 2022); and the Rey–Osterrieth Complex Figure Test (ROCF) for determining visuospatial constructional ability and visuospatial memory (Shin et al., 2006).

#### 2.3.2 Emotional evaluations

The Generalized Anxiety Disorder-7 (GAD-7), the Patient Health Questionnaire-9 (PHQ-9), and the Patient Health Questionnaire-15 (PHQ-15) were administered to measure anxiety, depression, and somatic symptoms, respectively, and exclusion cutoff scores were all set to 9 (Kroenke et al., 2001, 2002; Spitzer et al., 2006).

#### 2.3.3 ERP stimuli, recording, and preprocessing

**Figure 3:**
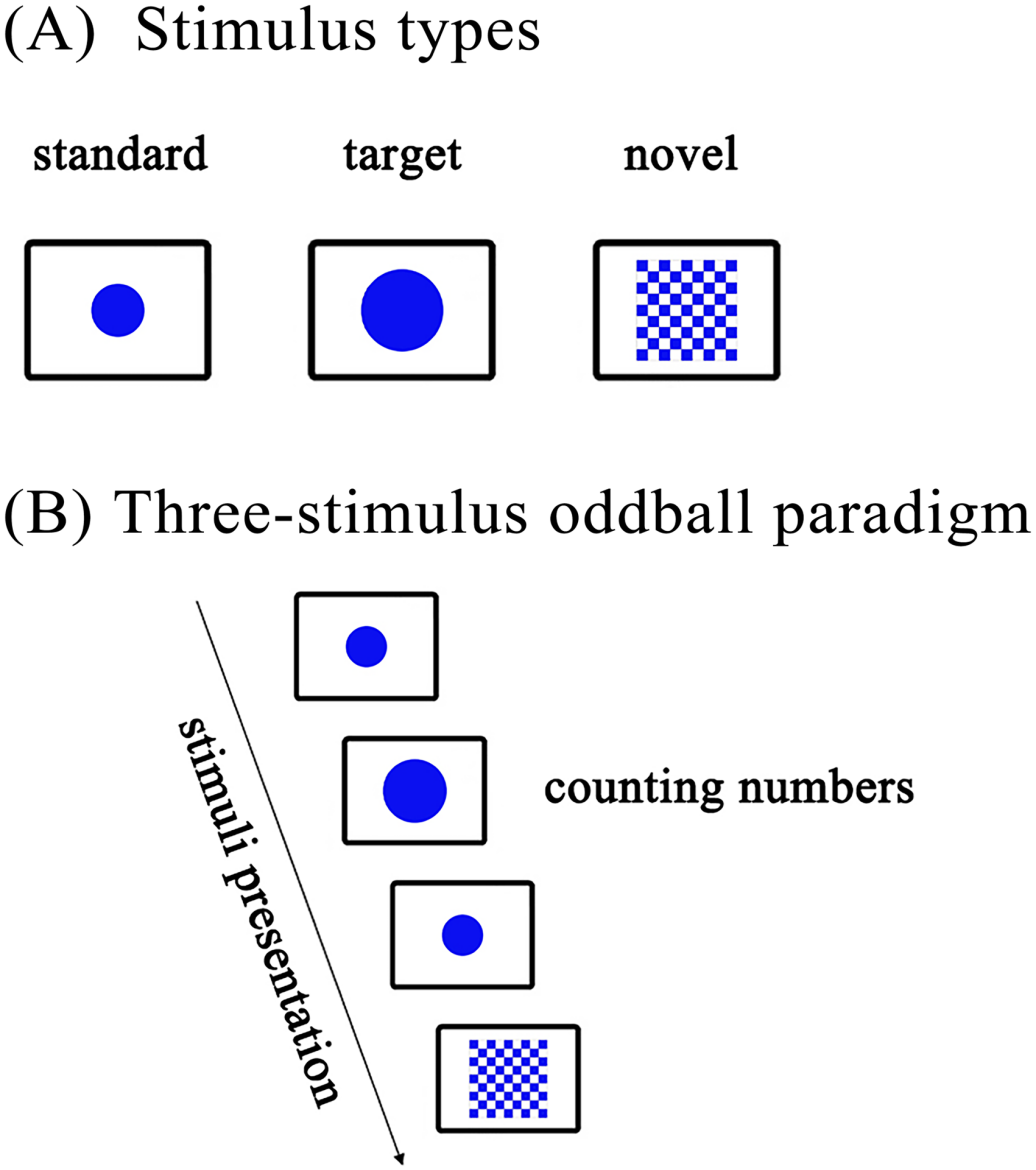
Schematic illustration of stimuli and paradigm. **(A)** Stimulus types. Stimuli include standard (smaller circles), target (larger circles), and novel (chessboards). **(B)** Three-stimulus oddball paradigm. Patients were instructed to discriminate and mentally count numbers of target stimuli.

Subjects were seated comfortably in an armchair inside an electrically shielded chamber and asked to fix their eyes on a monitor in front of them. A three-stimulus visual oddball paradigm was presented, and a total of 501 stimuli were displayed randomly in three blocks. Stimuli contained standard (smaller circles, *p* = 0.76), target (larger circles, *p* = 0.12), and novel (chessboards, *p* = 0.12) stimuli (Figure 3A), with durations and inter-stimulus intervals of 400 ms. Participants were instructed to identify and mentally count the number of target stimuli in each block (Figure 3B), and the accuracy was calculated based on the difference between the reported and actual numbers.

Electroencephalogram (EEG) signals were continuously recorded using Ag-AgCl electrodes mounted according to the 10–20 international system via a Neurolab EEG/ERP 32 Channel Amplifier (ANT Neuro, Netherlands). Recordings were obtained from midline Fz, Cz, and Pz, each referenced to the left mastoid signals. The electrode impedance was maintained below 5 kΩ. Electrooculogram (EOG) was recorded using electrodes placed above and below the right eye and 10 mm from the outer canthi. The sampling rate was 1,000 Hz, and the low-pass filter was 100 Hz.

ASA 4.9.3 software was used for analyzing EEG data. The data were re-referenced to the average of the bimastoid signals. EOG artifacts were removed using independent component analysis, followed by bandpass filtering (0.1–30 Hz, 24 dB/octave), epoch segmentations (200 ms pre-stimulus to 1,000 ms post-stimulus), and baseline corrections (−200–0 ms). Trials with drift or peak-to-peak deflection exceeding an ±100 μV amplitude were excluded from averaging. P3 (P3b, P3a) was defined as the largest positive peak between 300 and 600 ms. Peak amplitudes and latencies were then measured.

### 2.4 Definition of functional outcomes

The functional outcomes of symptomatic CSVD were assessed with the mRS at both admission and 90-day follow-up via face-to-face interviews and physical examinations. The mRS was scored from 0 to 6 with the following interpretations: 0–1 (no disability), 2–5 (increasing disability), and 6 (death). The absolute change in mRS score (ΔmRS) was calculated based on the difference between 90-day and baseline scores. Worse outcomes comprised neurological invariance (ΔmRS = 0) and deterioration (ΔmRS ≥ 1) (Jia et al., 2024; Polineni et al., 2025).

### 2.5 Statistical analysis

Continuous variables were presented as mean ± SD or median (interquartile range), and categorical variables were presented as frequencies (percentages). Normal distribution was checked using the Kolmogorov–Smirnov test. Continuous variables were evaluated using Student’s *t*-test or the non-parametric Mann–Whitney *U* test, whereas categorical variables were analyzed using the *χ^2^* test or Fisher’s exact test. The potential correlations between imaging markers and cognitive data were analyzed using exploratory Pearson’s (for continuous variables) or Spearman’s (for categorical variables) correlations. Fisher’s *r*-to-*z* comparisons were used to evaluate differences in correlation coefficients between the CSVD subgroups. Logistic regression analyses were constructed to identify independent predictors of poor functional outcomes. Variables with a *p-*value of ≤0.05 from the univariate analysis were included in the multivariate analysis to reduce the risk of overfitting. The bootstrap correction method was performed to internally validate models with 500 resamples (Wang et al., 2024). In addition, a pairwise receiver operating characteristic curve comparison between the models with PFD and models without PFD was performed to confirm its predictive value for functional outcomes. To ensure the stability and robustness of the constructed models, the intraclass correlation coefficient (ICC) or the kappa test was performed to assess inter-reader reproducibility of imaging markers. A two-sided *p-*value of <0.05 was considered significant. All statistical analyses were performed using SPSS 20.0 and GraphPad Prism 10.1.

## 3 Results

### 3.1 Baseline characteristics

A total of 126 patients were prospectively recruited, including 71 symptomatic CSVD patients (46 men) and 55 asymptomatic CSVD patients (37 men). As shown in Table 1, no significant difference was observed in demographic and clinical variables between the groups. Upon admission, symptomatic CSVD patients had NIHSS, mRS, and ADL scores of 3 (2–4), 2 (2–3), and 75 (60–85), respectively.

**Table 1:** Baseline characteristics and imaging markers of CSVD patients.

Variables/biomarkers	Symptomatic CSVD (*n* = 71)	Asymptomatic CSVD (*n* = 55)	*p*-values
Demographic and clinical			
Age, years	59.89 ± 9.95	61.18 ± 8.38	0.440
Male, *n* (%)	46 (64.8)	37 (67.3)	0.664
Education, years	12 (9–15)	12 (9–16)	0.131
BMI, kg/m^2^	26.47 ± 3.66	25.37 ± 3.38	0.086
Hypertension, *n* (%)	57 (80.3)	38 (69.1)	0.148
Diabetes mellitus, *n* (%)	26 (36.6)	17 (30.9)	0.503
Hyperlipidemia, *n* (%)	41 (57.7)	31 (56.4)	0.876
Coronary heart disease, *n* (%)	17 (23.9)	15 (27.3)	0.670
Current smoking, *n* (%)	29 (40.8)	18 (32.7)	0.350
Current alcohol consumption, *n* (%)	31 (43.7)	23 (41.8)	0.836
Medication usages			
Antiplatelet, *n* (%)	46 (64.8)	27 (49.1)	0.077
Statins, *n* (%)	56 (78.9)	42 (76.4)	0.737
Antihypertension, *n* (%)	51 (71.8)	34 (61.8)	0.234
Antidiabetic therapy, *n* (%)	26 (36.6)	16 (29.1)	0.374
Laboratory findings			
Triglyceride, mmol/L	1.23 (0.96–1.78)	1.26 (1.00–1.88)	0.898
Total cholesterol, mmol/L	4.41 ± 1.28	4.77 ± 1.11	0.101
HDL-C, mmol/L	1.26 ± 0.34	1.34 ± 0.39	0.272
LDL-C, mmol/L	2.63 ± 0.93	2.71 ± 0.81	0.633
Homocysteine, μmol/L	13.27 ± 4.71	12.10 ± 2.69	0.083
High-sensitivity CRP, mg/L	5.13 (2.26–8.15)	4.83 (1.83–7.15)	0.637
Impairment at admission			
NIHSS	3 (2–4)	–	–
mRS	2 (2–3)	–	–
ADL (Barthel Index)	75(60–85)	–	–

Continuous variables were expressed as mean ± standard deviation or median (interquartile range).

Categorical variables were expressed as *n* (%).

CSVD, cerebral small vessel disease; BMI, body mass index; HDL-C, high-density lipoprotein cholesterol; LDL-C, low-density lipoprotein cholesterol; CRP, C-reactive protein; NIHSS, National Institutes of Health Stroke Scale; mRS, Modified Rankin Scale; ADL, activities of daily living; WMHs, white matter hyperintensities; CMBs, cerebral microbleeds; EPVS, enlarged perivascular space; PFD, pericarotid fat density.

### 3.2 Imaging markers and cognitive data

The mean and maximum PFD values in symptomatic CSVD were higher than those in asymptomatic CSVD (all *p* < 0.001, Table 2). No significant difference was found between the groups regarding any CSVD markers, stenosis degree, calcification, ulceration, or maximum wall thickness. Excellent inter-reader agreements were obtained for CSVD markers and CTA variables (Supplementary Table S1).

**Table 2:** Imaging markers of CSVD patients.

Variables/biomarkers	Symptomatic CSVD (*n* = 71)	Asymptomatic CSVD (*n* = 55)	*p*-values
CSVD markers			
Lacunes, *n* (%)	40 (56.3)	27 (49.1)	0.419
Deep WMHs	2 (1–2)	2 (1–2)	0.127
Paraventricular WMHs	2 (2–2)	2 (1–2)	0.461
CMBs, *n* (%)	8 (11.3)	8 (14.5)	0.584
EPVS	2 (2–3)	2 (2–2)	0.340
CSVD total score	2 (2–3)	2 (1–3)	0.446
Carotid artery			
Stenosis degree			0.097
None, *n* (%)	7 (9.9)	10 (18.2)	
0–29%, *n* (%)	40 (56.3)	35 (63.6)	
30–49%, *n* (%)	24 (33.8)	10 (18.2)	
Calcification, *n* (%)	61 (85.9)	44 (80.0)	0.377
Maximum wall thickness, mm	3.01 (2.88–3.18)	2.92 (2.75–3.19)	0.164
Ulceration, *n* (%)	5 (7.0)	4 (7.3)	0.960
PFD values			
Mean PFD, HU	−62.05 ± 8.88	−67.82 ± 8.38	**<0.001** ^***^
Maximum PFD, HU	−60.07 ± 8.77	−65.89 ± 8.88	**<0.001** ^***^

Continuous variables were expressed as mean ± standard deviation or median (interquartile range).

Categorical variables were expressed as *n* (%). ***p < 0.001 by Student’s *t*-test. Bold values denote statistically significant associations (*p* < 0.05).

CSVD, cerebral small vessel disease; WMHs, white matter hyperintensities; CMBs, cerebral microbleeds; EPVS, enlarged perivascular space; PFD, pericarotid fat density.

In terms of cognitive function (Table 3), symptomatic CSVD patients exhibited poorer performance in multiple cognitive tests than asymptomatic CSVD patients, including MoCA, Stroop I, II, and III, ROCF copy, and recall (all *p* < 0.05). The ERP measures P3a and P3b amplitudes were significantly attenuated in the symptomatic group compared to the asymptomatic group (*p* = 0.017, *p* = 0.002). Other cognitive, behavioral, or emotional parameters did not reach significant levels (all *p* > 0.05).

**Table 3:** Cognitive and emotional data of CSVD patients.

Parameters	Symptomatic CSVD (*n* = 71)	Asymptomatic CSVD (*n* = 55)	*p*-values
Neuropsychological tests			
MoCA	25 (22–27)	26 (24–27)	**0.038** ^*^
STTA, s	16.76 ± 9.68	15.37 ± 8.09	0.391
STTB, s	33.62 (24.74–51.23)	25.77 (18.10–58.85)	0.065
Stroop I, s	31.00 (28.37–33.85)	29.41 (25.30–31.15)	**0.004** ^**^
Stroop II, s	22.32 (20.32–25.10)	20.82 (19.57–21.86)	**<0.001** ^***^
Stroop III, s	44.03 (39.94–49.68)	41.65 (38.02–43.43)	**0.010** ^*^
ROCF copy	33 (30–34)	34 (33–35)	**0.001** ^**^
ROCF recall	18.63 ± 4.19	20.35 ± 4.44	**0.029** ^*^
ERP tests			
accuracy, %	96.67 (95.00–100.00)	98.33 (96.67–100.00)	0.058
P3a amplitude, μV	6.02 ± 2.24	7.02 ± 2.35	**0.017** ^*^
P3b amplitude, μV	6.08 ± 2.28	7.49 ± 2.70	**0.002** ^**^
P3-standard amplitude, μV	1.21 ± 0.98	1.48 ± 1.23	0.188
P3a latency, ms	459.86 ± 44.20	453.52 ± 35.63	0.387
P3b latency, ms	464.59 ± 44.58	457.74 ± 34.61	0.349
P3-standard latency, ms	438.39 ± 41.78	435.82 ± 40.05	0.727
Emotional tests			
GAD-7	2 (1–4)	2 (0–4)	0.677
PHQ-9	3 (1–4)	3 (2–5)	0.179
PHQ-15	2 (0–4)	3 (1–5)	0.165

Continuous variables were expressed as mean ± standard deviation or median (interquartile range).

Categorical variables were expressed as *n* (%).

^*^p < 0.05, ^**^p < 0.01, ^***^p < 0.001 by student’s *t*-test or Mann–Whitney *U* test. Bold values denote statistically significant associations (*p* < 0.05).

CSVD, cerebral small vessel disease; MoCA, Montreal Cognitive Assessment; STT, Shape Trail Test; ROCF, Rey–Osterrieth Complex Figure Test; ERP, event-related potential; GAD-7, Generalized Anxiety Disorder-7; PHQ-9, Patient Health Questionnaire-9; PHQ-15, Patient Health Questionnaire-15.

### 3.3 Correlations between PFD and cognitive data

**Figure 4:**
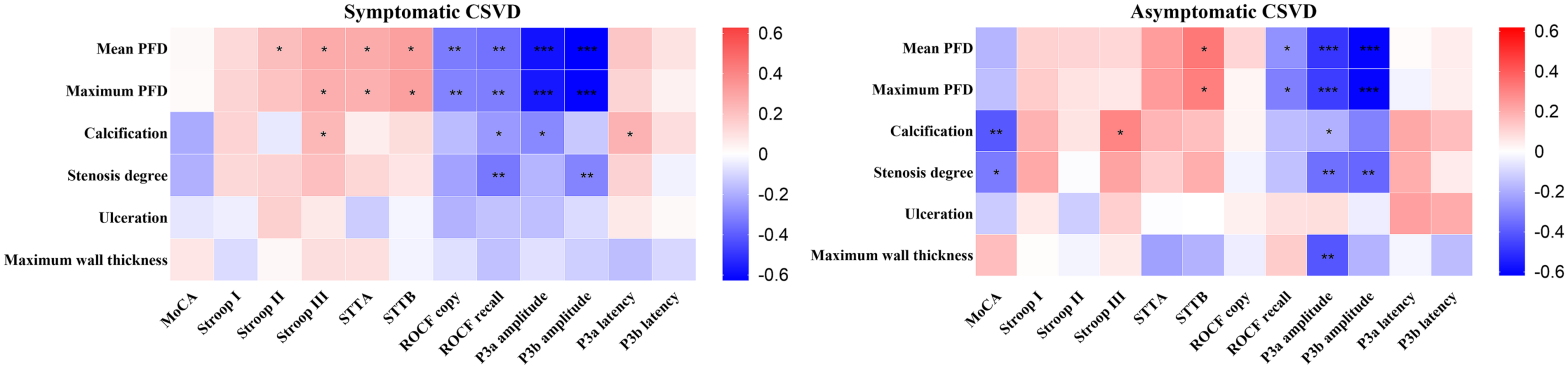
Correlation heatmaps between neuroimaging markers and cognitive data in symptomatic and asymptomatic CSVD. The bar’s color denotes correlation directions (positive: red; negative: blue), and color intensity denotes correlation strengths. ^*^p < 0.05, ^**^p < 0.01, ^***^p < 0.001 by exploratory Pearson’s or Spearman’s correlations. CSVD, cerebral small vessel disease; PFD, ericarotid fat density; MoCA, Montreal Cognitive Assessment; STT, Shape Trail Test; ROCF, Rey–Osterrieth Complex Figure Test.

The correlation coefficients between PFD values and cognitive performance in symptomatic CSVD were numerically larger compared with those in asymptomatic CSVD and involved more cognitive parameters (Figure 4, Supplementary Figure S1), although formal between-group comparisons only revealed significant differences in ROCF copy scores (Supplementary Tables S2, S3). In symptomatic CSVD patients, the mean and maximum PFD values were negatively correlated with ROCF copy/recall and P3a/P3b amplitude and positively correlated with Stroop III, STTA, and STTB (all *p* < 0.05). The mean PFD values were also correlated with reaction time in Stroop II (*p* = 0.042). Moreover, relatively few significant correlations between the mean/maximum PFD and cognitive data (STTB, ROCF recall, and P3a/P3b amplitude) were found in asymptomatic CSVD (all *p* < 0.05). The strongest correlations existed among ERP data in symptomatic CSVD (mean PFD: P3a amplitude: *r* = −0.587; P3b amplitude: *r* = −0.629; maximum PFD: P3a amplitude: *r* = −0.573; P3b amplitude: *r* = −0.623; all *p* < 0.001).

In addition to PFD values, other imaging markers, including calcification, stenosis degree, and maximum wall thickness, were associated with several cognitive parameters in symptomatic and asymptomatic CSVD groups, respectively, predominantly including MoCA (asymptomatic), Stroop III, ROCF recall (symptomatic), and P3a/P3b amplitude (all *p* < 0.05, Figure 4).

### 3.4 PFD performance in predicting adverse functional outcomes

In the symptomatic CSVD cohort, 21 patients (29.6%) suffered from adverse functional outcomes (ΔmRS ≥ 0). In univariate logistic regression models, a total of eight variables were associated with poor outcomes, including age, coronary heart disease, current smoking status, lacunes, CSVD total score, stenosis degree, mean PFD, and maximum PFD (all *p* ≤ 0.10). The multicollinearity analyses revealed a variance inflation factor value of 32.43 between the maximum PFD and the mean PFD. Consequently, only the maximum PFD rather than the mean PFD was included in multivariate logistic regression analyses considering its larger odds ratio (OR) value (Table 4, Supplementary Table S4). Lacunes, stenosis degree, and the maximum PFD (all *p* < 0.05) were entered into multivariate regression models to reduce the risk of overfitting. After multivariate adjustments, lacunes (OR: 3.190, 95% CI: 1.245–8.175, *p* = 0.016) and maximum PFD (OR: 8.879, 95% CI: 2.767–28.489, *p* < 0.001) were identified as independent predictors of adverse functional outcomes (Table 4).

**Table 4:** Multivariate analysis using maximum PFD in predicting poor functional outcomes.

Variables	Multivariate logistic regression analyses	
	OR (95% CI)	*p*-values
Lacunes	3.190 (1.245–8.175)	**0.016** ^*^
Stenosis degree	2.405 (0.996–5.812)	0.051
Maximum PFD	8.879 (2.767–28.489)	**<0.001** ^***^

Variables with *p* < 0.05 were included in multivariate logistic regression analyses.

^*^p < 0.05, ^***^p < 0.001. Bold values denote statistically significant associations (*p* < 0.05).

PFD, pericarotid fat density; OR, odds ratio; CI, confidence interval.

**Figure 5:**
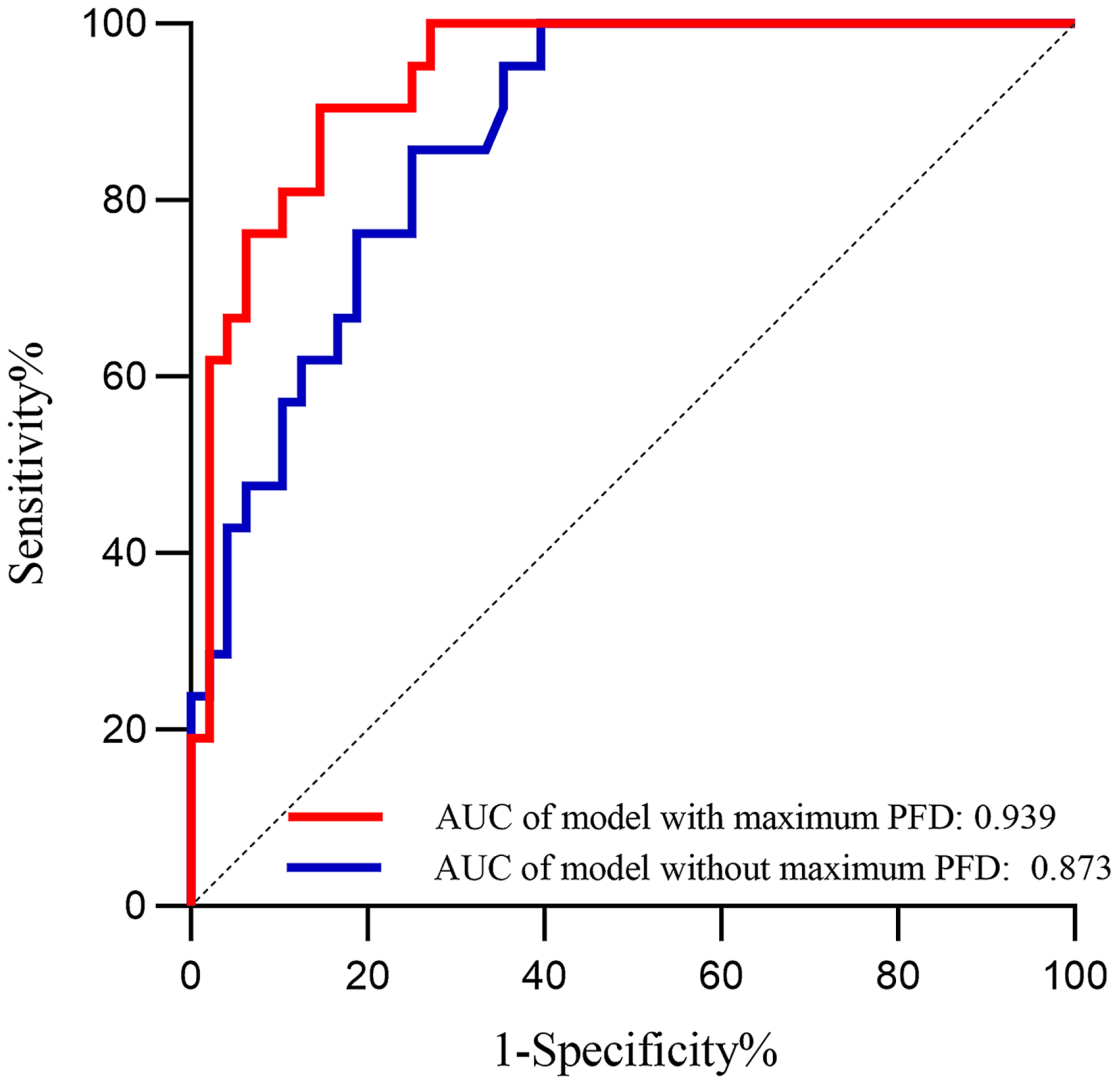
The AUC for predicting adverse functional outcomes (ΔmRS ≥ 0) in symptomatic CSVD. Variables in the model incorporating maximum PFD (red line) included age, coronary heart disease, current smoking status, lacunes, CSVD total score, stenosis degree, and maximum PFD. AUC, area under the curve; ΔmRS, Modified Rankin Scale change score; CSVD, cerebral small vessel disease; PFD, ericarotid fat density.

**Figure 6:**
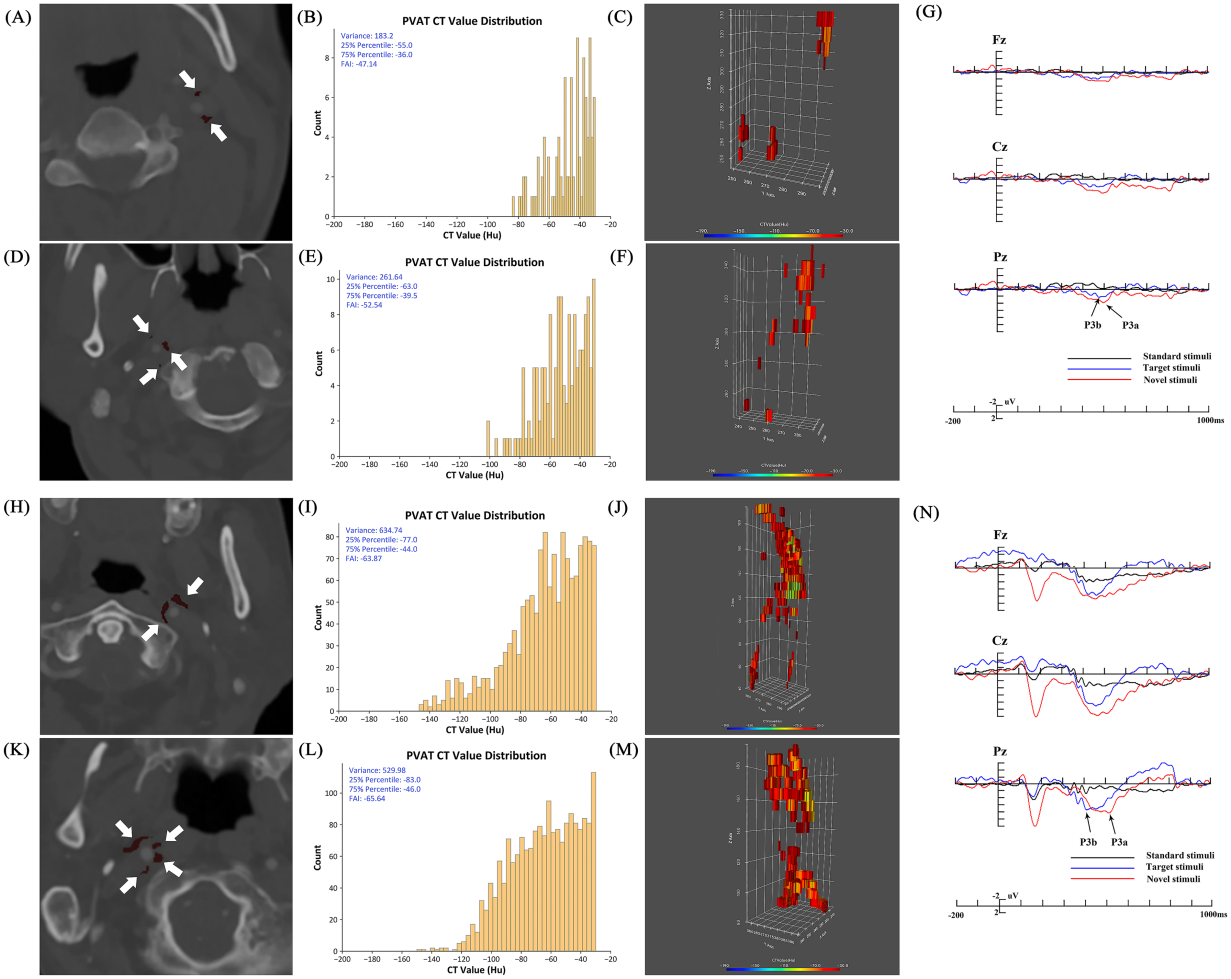
Representative cases of a 57-year-old male patient with symptomatic CSVD **(A–G)** and a 61-year-old male patient with asymptomatic CSVD **(H–N)**. Symptomatic CSVD exhibited higher PFD values than asymptomatic CSVD, with worse cognitive processing (attenuated P3a/P3b amplitude and prolonged P3a/P3b latency) and functional outcomes (ΔmRS = 2). PFD, pericarotid fat density; CSVD, cerebral small vessel disease; ERP, event-related potential; PVAT, perivascular adipose tissue; ΔmRS, Modified Rankin Scale change score.

The receiver operating characteristic curves were generated to determine the predictive efficacy of the maximum PFD. The area under the curve (AUC) for the maximum PFD was 0.873 (95% CI: 0.794–0.952, *p* < 0.001), with a sensitivity of 95.2% and a specificity of 74.0%. Symptomatic CSVD patients with maximum PFD values larger than −60.3 HU showed a high incidence of adverse clinical outcomes. The established models with PFD and those without PFD were compared to directly reflect its influence, and models incorporating maximum PFD (AUC = 0.939; 95% CI: 0.886–0.993) showed better performance than models without PFD (AUC = 0.873; 95% CI: 0.792–0.953) (Figure 5). After bootstrap correction, the AUCs of models with PFD reached 0.939 (95% CI: 0.882–0.987; Supplementary Figure S2). Figure 6 shows representative cases of PFD with distinct cognitive and functional outcomes.

## 4 Discussion

In this study, we revealed that an increased PFD level evaluated on carotid CTA was significantly correlated with cognitive impairment and unfavorable functional outcomes at 90 days in symptomatic CSVD. Compared with the asymptomatic group, numerically stronger associations between PFD values and cognitive data were found in the symptomatic group. The incorporation of PFD exhibited improved predictive performance in the constructed model for poor functional outcomes. These findings imply that PFD could serve as a novel prognostic biomarker to facilitate risk stratification and early targeted therapeutics in CSVD.

The novel biomarkers, mean and maximum PFD, was measured to characterize local perivascular inflammation. Since PVAT attenuation was negatively correlated with histological adipocyte size and the degree of adipocyte differentiation, with higher pericoronary tissue attenuation indicating lower lipid content in smaller adipocytes, the inflammation-induced alterations in PVAT and lipid metabolism imbalance could result in elevated PFD values on CTA (Antonopoulos et al., 2017; Xu et al., 2025). Although PET remains the gold standard for evaluating PVAT inflammation, its use is restricted due to high radiation exposure and expense (Mazurek et al., 2017). Routine CTA examination provides a non-invasive method to estimate localized perivascular inflammation and yields information comparable to PET (Antoniades et al., 2019), supporting the validity of our biomarkers for quantitative characterization of PVAT density.

Previous studies have predominantly focused on the roles of PFD in carotid plaque instability and cerebrovascular ischemic events (Baradaran et al., 2018; Zhang S. et al., 2022; Besler et al., 2024), whereas the associations between PFD and CSVD remain unclear. We found significantly increased mean/maximum PFD values in symptomatic CSVD patients compared with asymptomatic CSVD patients. Due to severe perivascular inflammation and high-risk plaque instability, acute infarcts were more likely to occur and cause clinical symptoms. Moreover, our retrospective study verified that higher PFD values were robustly associated with neuroimaging markers and adverse functional outcomes (mRS > 2) in symptomatic CSVD (Zhang et al., 2025). In our current prospective cohort, ΔmRS between follow-up and baseline was used to provide a dynamic change in functional impairment, and similar results were obtained regarding the predictive performance of PFD. Therefore, in a subset of CSVD patients with worse PFD, rigorous control of vascular risk factors (e.g., blood pressure <130/80 mmHg and low-density lipoprotein cholesterol <1.4 mmol/L), prompt drug treatment that might target PVAT (e.g., dual antiplatelet therapy, anti-inflammatory treatment, and vascular protection), and tailored rehabilitation should be performed to facilitate the prevention of sequelae and recurrence of infarcts (Cannistraro et al., 2019).

In addition to functional impairment, cognitive impairment caused by CSVD is considered an important precipitant of vascular dementia, with executive function and working memory being particularly affected (O'sullivan, 2010; Elahi et al., 2023). We combined sensitive ERP detection with a battery of neuropsychological scales to explore multidomain cognitive function (e.g., executive function, attention, visuospatial processing, and delayed memory) in CSVD patients. Compared with the asymptomatic group, the symptomatic group exhibited significantly worse performance in MoCA, Stroop, ROCF, and P3a/P3b amplitude, implying a tendency toward gradient cognitive decline and progressive deterioration in CSVD. More importantly, strong correlations between PFD values and cognitive data were observed, with more parameters involved in the symptomatic group. Our findings highlight that to improve cognitive prognosis, several drugs (e.g., cholinesterase inhibitors and dl-3-n-butylphthalide) might be used for patients with increased PFD levels (Peng, 2019), possibly beginning at the asymptomatic stage and intensifying at the more severe symptomatic stage.

Several mechanisms may explain the associations between PFD and cognitive/functional outcomes in CSVD. First, carotid PVAT acts as a potential hazardous biomarker for cerebral vessels, and impairments in the structure and function of the upstream large arteries can lead to hemodynamic disturbances in downstream small vessels, blood–brain barrier disruption, and vascular remodeling. These alterations in small vessels may accelerate the progression of CSVD lesions and ultimately result in cognitive decline and worse clinical outcomes (Zhai et al., 2020; Nyúl-Tóth et al., 2024). Second, carotid PVAT may secrete bioactive inflammatory mediators [e.g., tumor necrosis factor alpha (TNF-α), interleukin-1 (IL-1), and IL-6] that damage vascular walls and nearby tissues through paracrine mechanisms. The inflamed vessels can modulate PVAT phenotype and lipid accumulation, in turn, thereby forming vicious cycles. These inflammatory cascades induce oxidative stress, disrupt endothelial integrity, provoke chronic hypoperfusion in small perforating arteries, and aggravate parenchymal injury, which are closely associated with the pathogeneses and impairments in CSVD (Low et al., 2019; Cheng et al., 2023). Nevertheless, the exact roles of PVAT in CSVD deserve further elucidation, perhaps in animal models.

In line with previous studies on ischemic strokes (Yue et al., 2016; Wang et al., 2021), calcification and carotid stenosis degree also showed significant correlations with several cognitive parameters in symptomatic/asymptomatic CSVD. Arterial stiffness due to calcification may reduce arterial compliance and elevate pulse pressure. This hemodynamic disturbance attenuates resting cerebral blood flow, aggravates endothelial deficits, alters cerebral microcirculation, increases blood–brain barrier permeability, and leads to cognitive impairment (Muhire et al., 2019). Carotid stenosis contributes to chronic hypoperfusion in small vessels, microembolization, cerebrovascular reactivity impairment, and alterations in regional functional connectivity, which are principal mechanisms involved in the occurrence of ischemic lesions and the progression of cognitive impairment (Viticchi et al., 2021). In addition to PFD, lacunes emerged as a predictor of adverse functional outcomes in symptomatic patients. Regarded as the foci of former infarcts or hemorrhages within perforating arteries, lacunes are hypothesized to reflect brain aging/frailty, compromise cerebrovascular reserve, hinder post-stroke functional recovery, and disrupt cognitive cortical–subcortical networks (Benjamin et al., 2014; Zhou et al., 2022), hence resulting in poorer prognosis after acute infarcts or in symptomatic CSVD.

To the best of our knowledge, this was the first study to comprehensively explore the associations between PFD and outcomes in CSVD. The subclassification based on clinical symptoms, the combination of cognitive and functional outcomes, and the use of sensitive ERP measures were included as strengths of this study. Nevertheless, several limitations constrained the interpretation of our findings. First, as a single-center prospective study involving exploratory correlation analyses, our results should be interpreted cautiously and need to be externally validated in a larger multicenter cohort (usually with >500 participants); then the obtained PFD cutoff value or range could be more reliable in clinical practice. Second, cognitive data and ΔmRS score in symptomatic CSVD were merely evaluated at 90 days after infarcts, and an extended follow-up period should be performed to trace long-term outcome changes. Moreover, ΔmRS ≥0 was defined as adverse outcomes in symptomatic CSVD due to the majority of patients displaying mild functional impairments (mRS ≤ 2). The combination of mRS and ΔmRS scores and sensitivity analyses using alternative outcome definitions need to be performed in future cohort studies to further validate these findings. Finally, PFD was assessed only once at the baseline stage in the present study; hence, whether elevated PFD represented a stable chronic vascular feature or a transient inflammatory state following recent cerebrovascular events remained unclear. Longitudinal studies incorporating serially repeated PFD quantifications at multiple follow-up time points are required to resolve this distinction in future studies.

In conclusion, our study indicated that CTA-derived PFD was a promising predictor of cognitive impairment and adverse functional outcomes in symptomatic and asymptomatic CSVD. These findings may offer novel insights into prognosis prediction, risk-stratified prevention, and tailored therapy targeting PVAT in CSVD patients, even at an asymptomatic stage.

## Data Availability

The raw data supporting the conclusions of this article will be made available by the authors, without undue reservation.

## Supplementary material

SUPPLEMENTARY FIGURE S1 Scatter plots showing correlations between PFD and cognitive data. **(A)** Correlations between mean PFD and cognitive data in symptomatic CSVD (orange) and asymptomatic CSVD (blue) groups. **(B)** Correlations between maximum PFD and cognitive data in symptomatic CSVD (orange) and asymptomatic CSVD (blue) groups. ^*^*p* < 0.05, ^**^*p* < 0.01, ^***^*p* < 0.001 by exploratory Pearson’s or Spearman’s correlations. CSVD, cerebral small vessel disease; PFD, pericarotid fat density; MoCA, Montreal Cognitive Assessment; STT, Shape Trail Test; ROCF, Rey–Osterrieth Complex Figure Test.

SUPPLEMENTARY FIGURE S2 The bootstrap-corrected AUC for predicting adverse functional outcomes (ΔmRS ≥ 0) in symptomatic CSVD. Variables in the model incorporating maximum PFD (red line) included age, coronary heart disease, current smoking status, lacunes, CSVD total score, stenosis degree, and maximum PFD. AUC, area under the curve; ΔmRS, Modified Rankin Scale change score; CSVD, cerebral small vessel disease; PFD, pericarotid fat density.
