# Supplementary material for: Pericarotid fat density: a novel predictor of cognitive and functional outcomes in cerebral small vessel disease
Source: Front Aging Neurosci. 2026 Aug 3;18:1832953. doi: 10.3389/fnagi.2026.1832953 (PMC13478093; doi:10.3389/fnagi.2026.1832953)
Supplement: Supplementary file 1 [file Data_sheet_1.docx]

**Table S1 Inter-reader agreements analysis of CSVD markers and CTA variables**

| Variables | ICC value |
| --- | --- |
| mean PFD  maximum PFD | 0.966 (0.916, 0.986)  0.959 (0.905, 0.983) |
| maximum wall thickness | 0.894 (0.746, 0.958) |
| Variables | Kappa value |
| calcification  stenosis degree  ulceration | 0.959  0.929  0.902 |
| Lacunes  deep WMHs  paraventricular WMHs  CMBs | 0.921  0.878  0.895  0.816 |
| EPVS | 0.880 |
| CSVD total score | 0.889 |

Abbreviations: CSVD: cerebral small vessel disease, CTA: computed tomography angiography, PFD: pericarotid fat density, WMHs: white matter hyperintensities, CMBs: cerebral microbleeds, EPVS: enlarged perivascular space.

**Table S2 Comparisons of mean PFD-cognition correlations between CSVD subgroups**

| Cognitive parameters | *r* value | *Z* difference value | *P* value |
| --- | --- | --- | --- |
| MoCA |  |  |  |
| Symptomatic CSVD | 0.018 | 1.063 | 0.288 |
| Asymptomatic CSVD | -0.176 |  |  |
| STTA |  |  |  |
| Symptomatic CSVD | 0.275 | 0.215 | 0.830 |
| Asymptomatic CSVD | 0.238 |  |  |
| STTB |  |  |  |
| Symptomatic CSVD | 0.316 | -0.654 | 0.513 |
| Asymptomatic CSVD | 0.420 |  |  |
| Stroop I |  |  |  |
| Symptomatic CSVD | 0.125 | 0.340 | 0.734 |
| Asymptomatic CSVD | 0.063 |  |  |
| Stroop II |  |  |  |
| Symptomatic CSVD | 0.215 | 0.701 | 0.483 |
| Asymptomatic CSVD | 0.089 |  |  |
| Stroop III |  |  |  |
| Symptomatic CSVD | 0.277 | 1.365 | 0.172 |
| Asymptomatic CSVD | 0.033 |  |  |
| ROCF copy |  |  |  |
| Symptomatic CSVD | -0.325 | -2.562 | **0.010^*^** |
| Asymptomatic CSVD | 0.134 |  |  |
| ROCF recall |  |  |  |
| Symptomatic CSVD | -0.348 | -0.480 | 0.631 |
| Asymptomatic CSVD | -0.268 |  |  |
| P3a amplitude |  |  |  |
| Symptomatic CSVD | -0.587 | -0.751 | 0.453 |
| Asymptomatic CSVD | -0.489 |  |  |
| P3b amplitude |  |  |  |
| Symptomatic CSVD | -0.629 | -0.167 | 0.867 |
| Asymptomatic CSVD | -0.610 |  |  |
| P3a latency |  |  |  |
| Symptomatic CSVD | 0.187 | 0.978 | 0.328 |
| Asymptomatic CSVD | 0.009 |  |  |
| P3b latency |  |  |  |
| Symptomatic CSVD | 0.091 | 0.262 | 0.793 |
| Asymptomatic CSVD | 0.043 |  |  |

^*^*P* < 0.05 by *Fisher r-to-z* comparisons.

Abbreviations: PFD: pericarotid fat density; MoCA: Montreal Cognitive Assessment; STT: Shape Trail Test; ROCF: Rey-Osterrieth Complex Figure Test.

**Table S3 Comparisons of maximum PFD-cognition correlations between CSVD subgroups**

| Cognitive parameters | *r* value | *Z* difference value | *P* value |
| --- | --- | --- | --- |
| MoCA |  |  |  |
| Symptomatic CSVD | 0.016 | 0.946 | 0.344 |
| Asymptomatic CSVD | -0.157 |  |  |
| STTA |  |  |  |
| Symptomatic CSVD | 0.258 | 0.087 | 0.931 |
| Asymptomatic CSVD | 0.243 |  |  |
| STTB |  |  |  |
| Symptomatic CSVD | 0.316 | 0.060 | 0.952 |
| Asymptomatic CSVD | 0.306 |  |  |
| Stroop I |  |  |  |
| Symptomatic CSVD | 0.139 | 0.373 | 0.709 |
| Asymptomatic CSVD | 0.071 |  |  |
| Stroop II |  |  |  |
| Symptomatic CSVD | 0.195 | 0.811 | 0.950 |
| Asymptomatic CSVD | 0.048 |  |  |
| Stroop III |  |  |  |
| Symptomatic CSVD | 0.264 | 1.289 | 0.198 |
| Asymptomatic CSVD | 0.033 |  |  |
| ROCF copy |  |  |  |
| Symptomatic CSVD | -0.306 | -2.206 | **0.028^*^** |
| Asymptomatic CSVD | 0.090 |  |  |
| ROCF recall |  |  |  |
| Symptomatic CSVD | -0.319 | -0.048 | 0.962 |
| Asymptomatic CSVD | -0.311 |  |  |
| P3a amplitude |  |  |  |
| Symptomatic CSVD | -0.573 | -0.749 | 0.454 |
| Asymptomatic CSVD | -0.473 |  |  |
| P3b amplitude |  |  |  |
| Symptomatic CSVD | -0.623 | -0.105 | 0.916 |
| Asymptomatic CSVD | -0.611 |  |  |
| P3a latency |  |  |  |
| Symptomatic CSVD | 0.139 | 0.922 | 0.356 |
| Asymptomatic CSVD | -0.030 |  |  |
| P3b latency |  |  |  |
| Symptomatic CSVD | 0.046 | 0.038 | 0.970 |
| Asymptomatic CSVD | 0.039 |  |  |

^*^*P* < 0.05 by *Fisher r-to-z* comparisons.

Abbreviations: PFD: pericarotid fat density; MoCA: Montreal Cognitive Assessment; STT: Shape Trail Test; ROCF: Rey-Osterrieth Complex Figure Test.

**Table S4 Multivariate analysis using mean PFD in predicting poor functional outcomes**

| Variables | Multivariate logistic regressions | |
| --- | --- | --- |
|  | OR (95% CI) | *P* values |
| Lacunes | 3.187 (1.289-7.883) | **0.012^*^** |
| Stenosis degree | 2.157 (0.896-5.192) | 0.086 |
| Mean PFD | 7.291 (2.535-20.973) | **< 0.001^***^** |

Variables with *P* < 0.05 were included in multivariate logistic regressions.

^*^*P* < 0.05, ^***^*P* < 0.001.

Abbreviations: PFD: pericarotid fat density, OR: odds ratio, CI: confidence interval.
